# ABCDE of prehospital ultrasonography: a narrative review

**DOI:** 10.1186/s13089-018-0099-y

**Published:** 2018-08-08

**Authors:** Rein Ketelaars, Gabby Reijnders, Geert-Jan van Geffen, Gert Jan Scheffer, Nico Hoogerwerf

**Affiliations:** 1 Radboud Institute for Health Sciences, Department of Anesthesiology, Pain, and Palliative Medicine, Radboud university medical center, Geert Grooteplein-Zuid 10, 6525 GA Nijmegen, The Netherlands; 2 Radboud Institute for Health Sciences, Helicopter Emergency Medical Service Lifeliner 3, Radboud university medical center, Geert Grooteplein-Zuid 10, 6525 GA Nijmegen, The Netherlands; 3 Department of Intensive Care, Catharina Hospital, Michelangelolaan 2, 5623 EJ Eindhoven, The Netherlands

**Keywords:** Prehospital, Ultrasonography, Diagnostic imaging, Emergency medical services, Air ambulances, Emergency medicine, Review

## Abstract

Prehospital point-of-care ultrasound used by nonradiologists in emergency medicine is gaining ground. It is feasible on-scene and during aeromedical transport and allows health-care professionals to detect or rule out potential harmful conditions. Consequently, it impacts decision-making in prioritizing care, selecting the best treatment, and the most suitable transport mode and destination. This increasing relevance of prehospital ultrasonography is due to advancements in ultrasound devices and related technology, and to a growing number of applications. This narrative review aims to present an overview of prehospital ultrasonography literature. The focus is on civilian emergency (trauma and non-trauma) setting. Current and potential future applications are discussed, structured according to the airway, breathing, circulation, disability, and environment/exposure (ABCDE) approach. Aside from diagnostic implementation and specific protocols, procedural guidance, therapeutic ultrasound, and challenges are reviewed.

**Electronic supplementary material:**

The online version of this article (10.1186/s13089-018-0099-y) contains supplementary material, which is available to authorized users.

## Introduction

Point-of-care ultrasound (PoCUS) refers to a sign or symptom-based ultrasonography (US) examination either at the bedside or wherever patients are being treated [1]. The use of PoCUS by nonradiologists is being adopted in prehospital emergency care. It may help health-care professionals of emergency medical services (EMS) to diagnose or rule out potential life-threatening or otherwise harmful conditions [2–4]. Prehospital point-of-care ultrasonography (PHUS) may have an impact on decision-making in prioritizing initial treatment and choosing the most appropriate hospital and mode of transportation [5, 6]. Besides deploying PHUS for diagnostic purposes, it is used for procedural and therapeutic interventions.

Although the use of PHUS is increasing, its added value is still under debate. In 2010, Jørgensen was unable to conclude that PHUS improves treatment of trauma patients [5]. Five years later, O’Dochartaigh found only moderate evidence to support the use of PHUS in physician-staffed prehospital systems [6]. A recent Cochrane review concluded that, at best, abdominal US has no negative impact on mortality and morbidity, although it might reduce ordered computed tomography (CT)-scans [7]. Rudolph et al. found that PHUS may improve patient management with respect to diagnosis, treatment, and hospital referral [8]. However, they were unable to assess the effect of PHUS on patient outcomes based on the current body of evidence.

The image quality, size, and weight of portable ultrasound devices are improving. Costs for equipment are decreasing, while the number of indications for PHUS is increasing. The result is an exponentially growing body of publications, including some narrative reviews, with varying perspectives [9–11].

This narrative review is based on relevant papers selected from an extensive search in the Ovid MEDLINE^®^ database. We added additional papers found in the references and from the authors’ personal libraries.

The aim is to present an overview of the literature on PHUS in a civilian emergency (trauma and non-trauma) setting. The first part deals with current PHUS applications structured according to the familiar airway, breathing, circulation, disability, and exposure/environment (ABCDE) approach [12]. The second part will discuss interventions, procedures, challenges, and potential future applications.

## PHUS in general

The use of PHUS provides diagnostic and therapeutic benefit, and it does not delay patient management [3, 8, 13–15]. It has been found to be feasible to enhance clinical assessment in a variety of out-of-hospital settings [15]. Price was among the first to show that ultrasonography (US) is also feasible during helicopter transport and that focused assessment with sonography for trauma (FAST) can be rapidly performed in-flight and has no influence on aircraft avionics [16].

Physicians and paramedics without being educated as a radiologist can be trained effectively to perform PoCUS. Lyon et al. demonstrated that prehospital critical care providers could learn to detect the sonographic sliding lung sign with a high level of sensitivity (97%) and specificity (94%) and retain their skill over time [17]. Forty physicians participated in a 4-h hands-on training and demonstrated significant improvements in the ability to perform US examinations [18]. Although the initial learning curve for FAST is steep, it starts to flatten after 30–100 scans [19]. Probably even more training and experience is required for advanced applications such as transcranial Doppler for ischemic stroke or specific triage protocols.

With the right education and mentorship, paramedics can obtain ultrasound images of sufficient quality to positively identify significant pathologies in critically ill patients [20]. A recent Canadian study found that PHUS performed by both physicians and non-physicians supported interventions in both trauma and medical patients [21].

The reported diagnostic accuracy of PHUS varies widely. Some reported a sensitivity of 85–90% and a specificity of 96–100% for chest, abdominal, and cardiac US. Positive predictive value (PPV) and negative predictive value (NPV) were 100 and 95.5% [3, 13]. Diagnostic accuracy during transportation also varies. For PHUS during transfer by ground ambulance and PHUS on-scene, Brun reported a sensitivity of 94.7 and 95.2%, respectively [22]. In-flight ultrasound examinations of the lung, abdomen, and pericardium yielded a sensitivity of only 50–64.7% when compared to pathology that required an intervention, rather than to all positive findings [23]. Others found a sensitivity of 78.6% for in-flight extended FAST (eFAST) compared to CT scan [24]. Because of the high specificity, the activation of a trauma surgery team is justified for positive PHUS findings [23].

Despite the range in diagnostic accuracy, PHUS is still highly reliable compared to clinical assessment [3, 5]. In 169 non-trauma patients, PHUS improved the diagnostic accuracy based on traditional clinical examination to 67% compared to the final in-hospital diagnosis. Diagnostic accuracy was improved in 90% of patients in whom the initial diagnosis was uncertain (n = 115) [25]. Blaivas found that PHUS improved the certainty of the diagnosis in 68% of 25 mainly non-trauma patients [26].

PHUS potentially impacts life-saving procedures, priorities in the care for one or many patients, and the most appropriate destination. Indications exist that PHUS benefits outcome, but evidence is still lacking [5]. Nevertheless, O’Dochartaigh and Jørgensen noted that PHUS impacted and streamlined in-hospital treatment [5, 6]. The impact was not quantified, but O’Dochartaigh suggested that PHUS-supported interventions were more frequent in the more severely injured patients.

## Diagnostic applications

### A—Airway

First-attempt success rates of prehospital rapid sequence intubations vary between 46 and 85% [27]. An attempt fails when the endotracheal tube (ETT) cannot be placed between the vocal cords in the trachea or is inadvertently placed in the esophagus. It is of paramount importance to acknowledge esophageal intubation as soon as possible.

The use of tracheal and cricothyroid ultrasound can be very useful to confirm correct ETT placement. This was first described in neonates by Slovis in 1986 [28]. Fourteen years later, Dreschler was the first to also visualize the esophagus and to detect esophageal intubation in five out of five cadaver models [29]. A recent review showed a pooled sensitivity and specificity of, respectively, 98 and 94% of transtracheal US in emergency intubations [30]. Therefore, the confirmation of correct ETT position by PoCUS in the prehospital setting is likely to be beneficial [12, 31, 32]. Although capnography is considered the gold standard to confirm a correct tube position, it does not discriminate between endotracheal and endobronchial intubation [33]. Furthermore, in a prehospital setting, chest radiography is impossible, and auscultation is not always feasible. Therefore, PHUS might be a valuable tool to assess the airway [34].

Zadel et al. confirmed endotracheal tube position by the detection of bilateral lung sliding and bilateral diaphragmatic excursion in 124 out-of-hospital patients [32]. Esophageal intubation occurred in 13 patients (10.5%), of which only 30% was detected visually or by auscultation before waveform capnography was recorded. Both sensitivity and specificity of PHUS for a correct tube position was 100%. The performance of PHUS took a median of 30 s (sd = 8–120 s). A prospective study in pediatrics preferred the assessment of bilateral diaphragmatic excursions to confirm proper ETT placement [35]. Therefore, the assessment of lung sliding and diaphragmatic excursions is of value in the absence of chest radiography or capnography.

### B—Chest, pulmonary

The cause of acute dyspnea is not immediately apparent, especially in the prehospital setting. Caregivers must differentiate between a cardiac or a pulmonary cause. In an emergency department (ED) study, Kajimoto proposed a quick method to integrate (1) lung ultrasound, (2) cardiac ultrasound, and (3) measurements of the inferior vena cava (LCI) [36]. Lung ultrasound is performed in eight chest areas (four anterior and four lateral). Cardiac ultrasound estimates the global left ventricular function and mitral or tricuspid valve regurgitation. Subsequently, collapsibility of the inferior vena cava is determined. The LCI integrated examination will take only up to 3 min. The sensitivity and specificity were 94.3 and 91.9% for acute heart failure syndromes, compared to the traditional methods of differentiating between pulmonary and cardiac causes including electrocardiogram, chest X-ray, and B-type natriuretic peptide (BNP).

A similar *triple scan* consisting of basic echocardiography, lung ultrasound, and assessment of inferior vena cava collapsibility was proposed by Mantuani et al. [37]. They included 57 patients with acute dyspnea caused by acute decompensated heart failure (ADHF), chronic obstructive pulmonary disease (COPD), and pneumonia. After the triple scan, the accuracy of the diagnosis, based on history and physical examination, increased from 53 to 77%. Sensitivity and specificity of the triple scan for ADHF were 100 and 84%.

Lichtenstein’s bedside lung ultrasound in emergency (BLUE) protocol allows rapid diagnosis of acute respiratory failure and can be completed in under 3 min [38, 39]. Four standardized points on either side of the chest are assessed for ten signs indicative of normal lung surface, pleural effusions, lung consolidations, alveolar–interstitial syndrome, and pneumothorax. For simplicity, echocardiography is not included. Distinct profiles are recognized for the main causes of respiratory distress: pneumonia, congestive heart failure, COPD, asthma, pulmonary embolism, and pneumothorax as summarized in Table 1. It has a diagnostic accuracy of > 90% [40].

**Table 1:** BLUE protocol profiles. Lichtenstein [39]

P#	Profile name	Location	Appearance	Implication/diagnosis
1	A-profile	Anterior chest wall	Lung sliding—visualization of the movement of the visceral pleura against the parietal pleura with respiration A-lines—an indication of the presence of air below the parietal pleura^a^	Normal lung surface
2	B-profile		Lung sliding Lung rockets—a pattern of three vertical B-lines caused by edema in the interlobular septa^b^	Pulmonary edema
3	B’-profile		No lung sliding—in the B’ profile lung sliding is abolished by the deposition of fibrin caused by pneumonia Lung rockets	Pneumonia
4	A/B-profile		Unilateral lung rockets—indicative for a (unilateral) pneumonia and does not correspond with generalized pulmonary edema	Pneumonia
5	C-profile	Anterior chest wall	Anterior lung consolidation—anteriorly located, therefore unlike to be caused by hemodynamic pulmonary edema or embolism	Pneumonia
6	A-profile without DVT^c^		Lung sliding A-lines^a^ No DVT	Normal
	A-no-V-PLAPS profile	Posterolateral chest wall	Lung sliding A-lines^a^ No DVT PLAPS^e^—posterolateral alveolar and/or pleural syndrome—pulmonary consolidation and pleural effusion	Pneumonia
7	A-profile with DVT^c^		Lung sliding A-lines^a^ DVT	Pulmonary embolism
8	A’-profile	Anterior chest wall	No lung sliding—lung sliding abolished by separation of the visceral pleura from the parietal pleura A-lines—an indication of the presence of air below the parietal pleura^a^	Pneumothorax when the mandatory ‘lung point’^d^ is visualized
9	A-profile without DVT and no PLAPS (nude profile)		Lung sliding A-lines^a^ No DVT No PLAPS	Asthma or COPD

The bedside lung ultrasound in emergency (BLUE) protocol defines nine profiles. They are defined by their sonographic appearance and are associated with the different diagnoses as described in the right-most column

^a^A-lines = Horizontal repetition of the pleural line appearing below the pleural line at multiples of the skin–pleural line distance. Their appearance is an indication of air below the parietal pleura, either in or outside of the lung. They are particularly apparent in the absence of B-lines potentially obscuring the A-lines

^b^B-lines = A long, well-defined, hyperechoic comet tail artifact arising from the pleural line that obliterates the A-lines

^c^DVT= Deep venous thrombosis. Has to be separately found or excluded at the lower extremities

^d^Lung point = The location where the visceral pleura is only partially in contact with the parietal pleura. With respirations, the A’ profile (without lung rockets) is intermittently replaced with the A-profile (lung rockets are possible). The lung point is a pathognomonic sign for the diagnosis of pneumothorax! See Additional file 1: Video 1

^e^PLAPS= Posterolateral alveolar and/or pleural syndrome (posterolateral consolidations or pleural effusions)

The LCI, *triple scan*, and BLUE protocol all might be relevant and valuable in the prehospital setting because of simplicity and nominal time investment. With the help of these protocols, the EMS caregiver can accurately differentiate between causes and direct treatment and avoid unnecessary or harmful interventions.

**Fig. 1:**
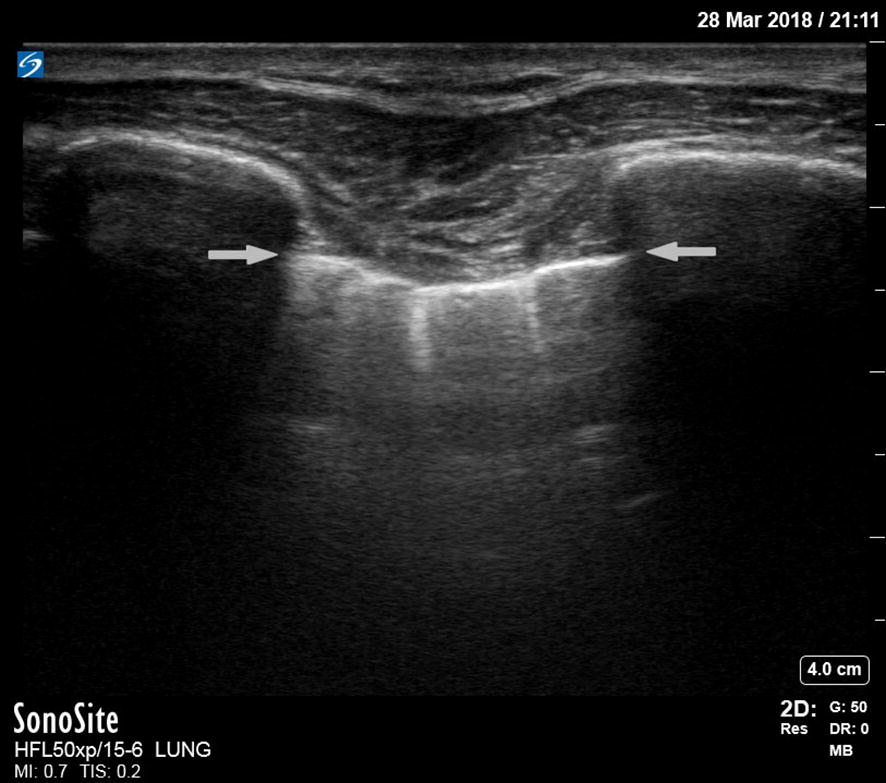
Normal lung. A normal lung ultrasound image acquired with a 15–6 MHz linear transducer. The ribs are visible with their anechoic shadows on both sides of the image. The pleural line is shown in between the ribs, indicated with two horizontal arrows. Emanating down from the pleural line are comet tails. B-lines (not visible here) also start at the pleural line, but extend all the way down to edge of the image

**Fig. 2:**
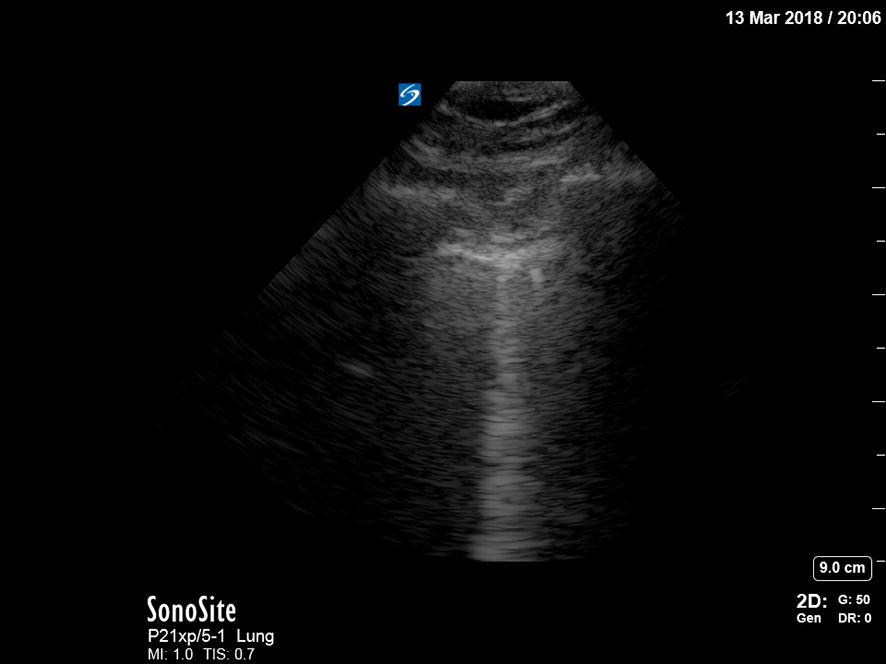
Normal lung + B-line. A normal lung ultrasound image acquired with a 5–1 MHz phased-array cardiac transducer. In the middle of the image, one B-line is seen. This is also seen in healthy subjects and a single B-line is without meaning

Besides the diagnosis of dyspnea, lung US may be used to support prehospital continuous positive airway pressure (CPAP) treatment [41]. In 20 ADHF patients, a physician-staffed EMS sonographically assessed 15 chest wall regions before and after CPAP treatment compared to standard treatment. The number of B-lines (explained in Table 1 and shown in Figs. 1, 2) was significantly lower in the CPAP group, and their respiratory and hemodynamic variables improved after CPAP. The number of B-lines correlates with the amount of extravascular lung water (EVLW) and NT-proBNP levels and thus with the severity of ADHF. They develop at a pulmonary artery occlusion pressure (PAOP) > 18 mmHg [42].

In high-altitude medicine, Wimalasena described the value of lung US in the early detection of high-altitude pulmonary edema (HAPE) before symptoms appear, and in differentiating HAPE from other causes of dyspnea such as pneumonia or pneumothorax [43]. The number of B-lines, indicating an increased amount of EVLW, is inversely correlated with the oxygen saturation and both values improve on (early) treatment [44].

### B—Chest, traumatic

#### Pneumothorax

Using PoCUS for detecting pneumothorax is feasible, fast, without any radiation, has a steep learning curve and high diagnostic accuracy, and it allows for dynamic and repeated examinations [45, 46].

**Fig. 3:**
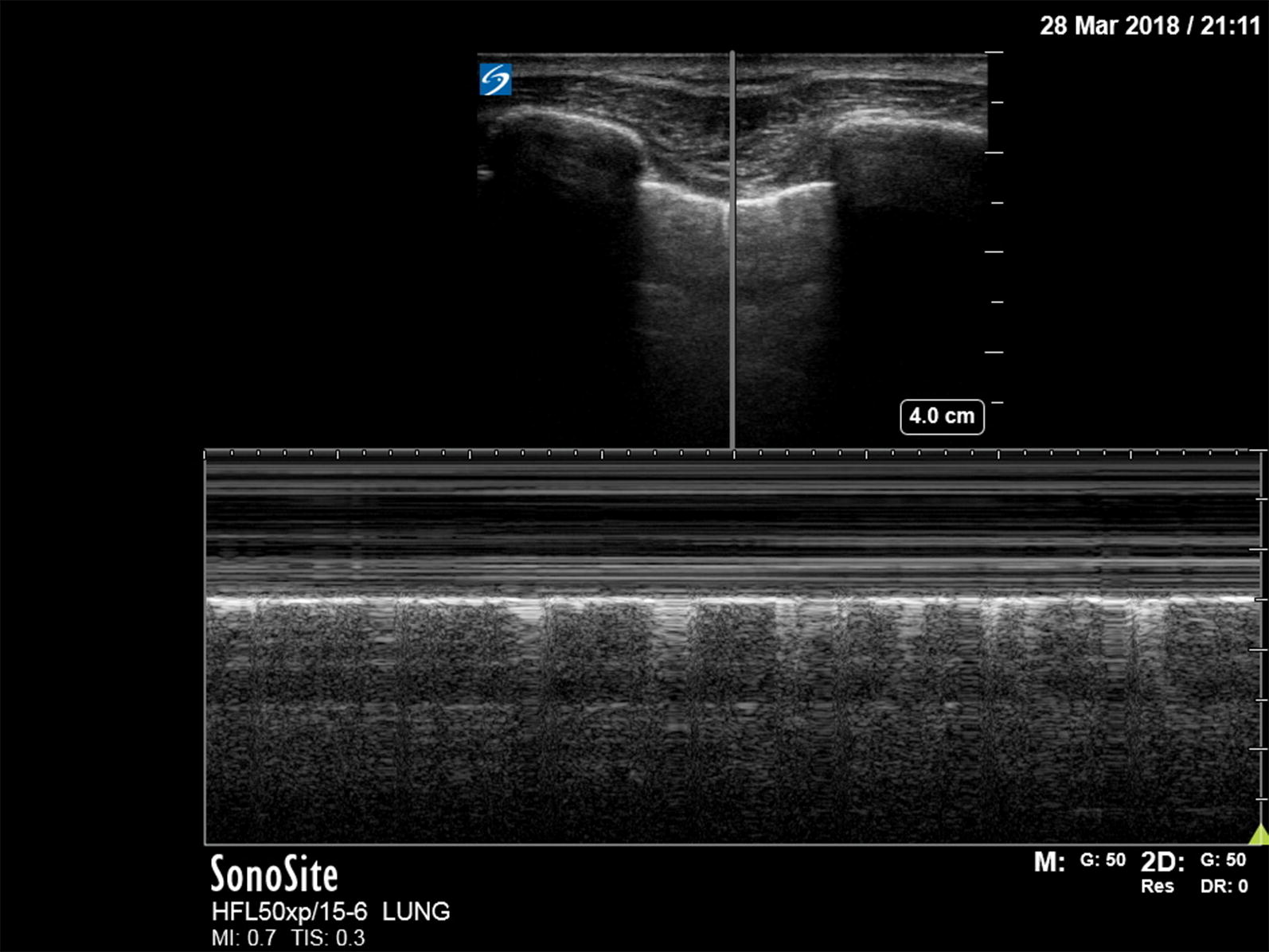
Normal lung—M-mode ultrasound image. The ultrasound reflections on the vertical line in the upper part of the image are sequentially displayed from left to right in the lower part as time progresses. It allows to capture the motion of the upper 2D image in the stationary image below. A normal M-mode image of the chest wall and pleura is displayed here. The stationary chest wall produces straight horizontal lines above the pleural line. The lung sliding and movement of the artifacts below produces a grainy image. This is called the seashore sign

**Fig. 4:**
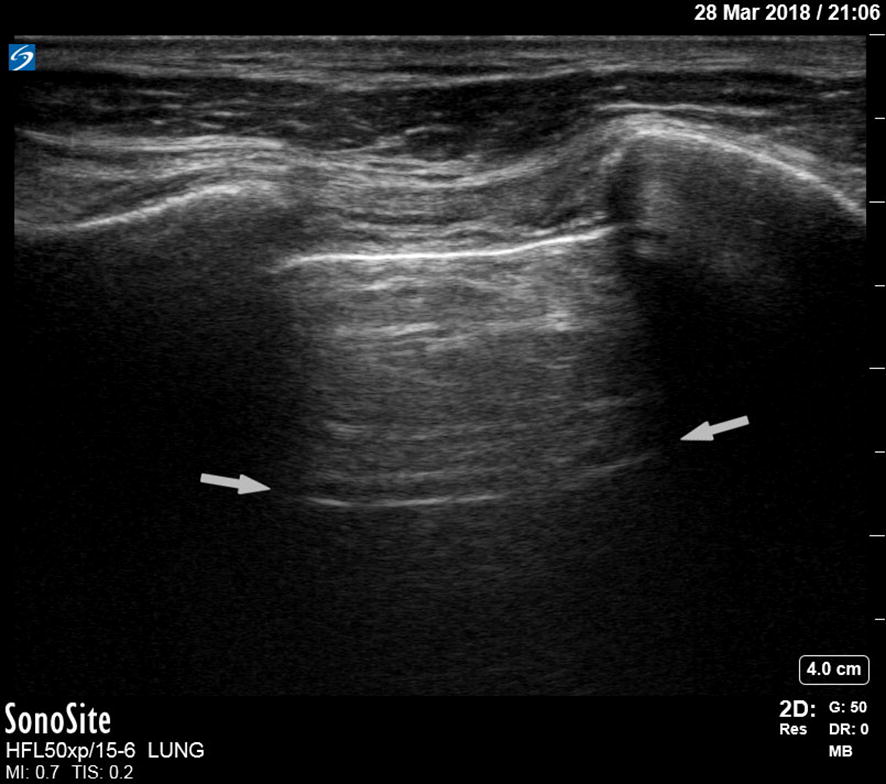
Pneumothorax + A-line. A-lines are reflections of the pleural line caused by gas below the parietal pleura. An A-line is indicated by the arrows. The A is for “air” either within or outside of the lung. In case of a pneumothorax, there are no B-lines (Fig. 2) that may obscure the A-lines making them stand out more clearly

**Fig. 5:**
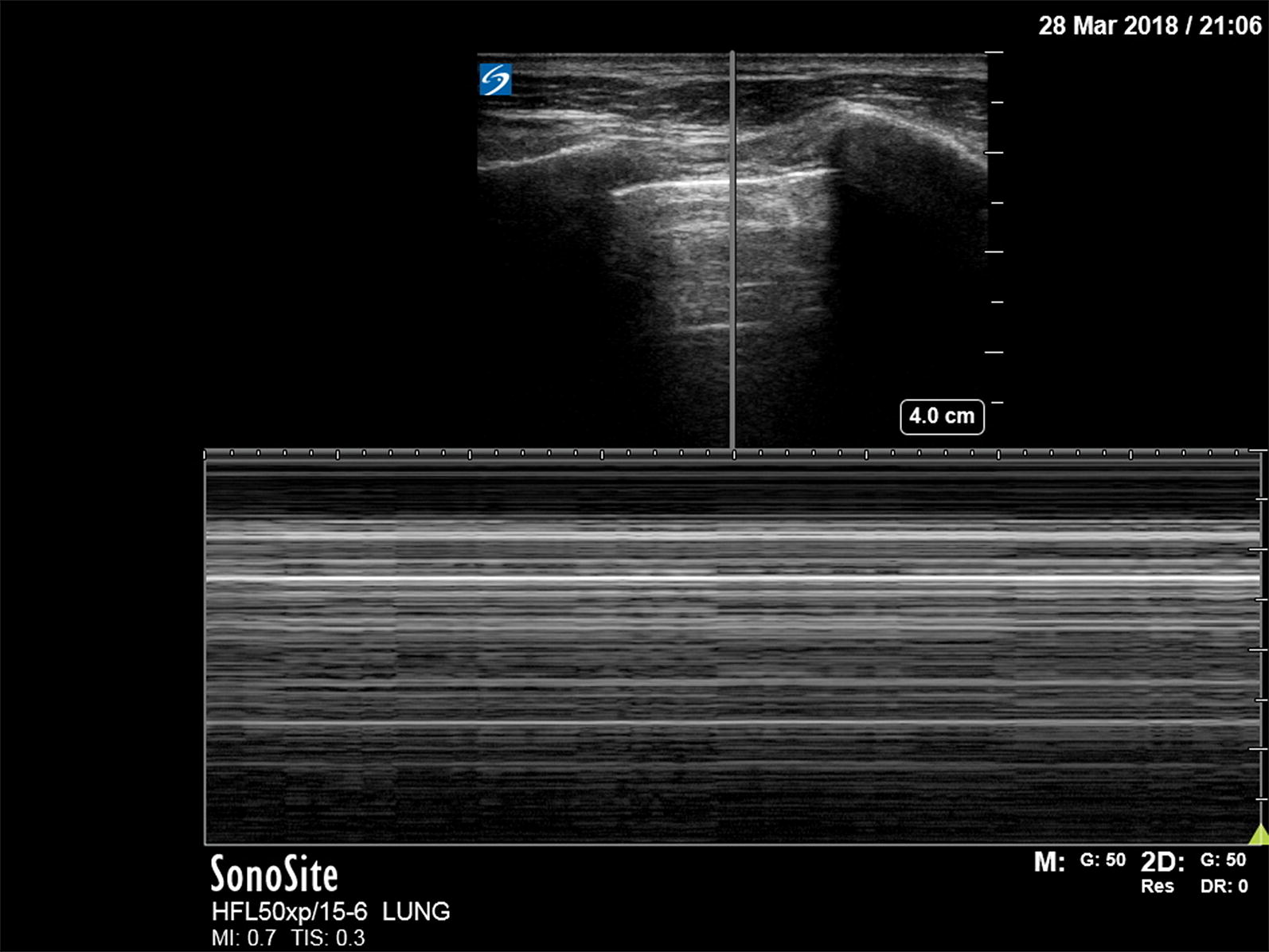
Pneumothorax—M-mode ultrasound image. There is no visible movement above or below the pleural line. Because all tissue and artifacts are stationary, the M-mode image appears as horizontal straight lines throughout the image. This is called a barcode sign or stratosphere sign

A pneumothorax is characterized by the abolition of lung sliding, the absence of B-lines, and the appearance of the A-line sign [39]. Lung sliding is the representation of the visceral and parietal pleura sliding against each other during respiration. B-lines are the result of the accumulation of fluid in the pulmonary interstitium. Therefore, the presence of B-lines on PoCUS rules out a pneumothorax. Horizontal A-lines are reflections of the pleural line caused by gas below the parietal pleura either within or outside of the lung. These signs are explained further in Table 1; adapted from Lichtenstein’s paper on the BLUE and FALLS protocol [39]. A normal lung US image is shown in Figs. 1, 2, and 3. Images of pneumothorax are shown in Figs. 4, 5, and Additional file 1: Video 1.

An important implication of detecting or excluding pneumothorax is the subsequent decision to perform (or withhold) a tube thoracostomy. An evaluation of prehospital chest US in 281 patients revealed that the acute medical management changed in 21%. The intention to introduce a tube thoracostomy was abandoned in 4% (n = 10) and the transport destination changed in another 4% [4]. Similarly, Mazur found that chest US examinations (n = 60) performed in preparation for air transport helped them prevent four (8%) chest tube thoracostomies [47].

#### Detection of pneumothorax during flight

In some EMS systems, patients are evaluated and treated while in-flight in a helicopter or fixed-wing aircraft. In prehospital and flight medicine, lung US was found to be feasible and safe [16, 24, 48]. For instance, M-mode (Figs. 3, 5) ultrasonography was used to successfully evaluate a pleural interface model on board a helicopter while stationary, with rotor rotation before take-off, and at level flight [49]. Madill reported the case of a blast injury patient in whom an in-flight chest US examination identified an untreated pneumothorax. This directed the decision to perform a successful in-flight thoracentesis and tube thoracostomy [50]. In 2013, Roline was reported to be the first to evaluate in-flight chest US in a helicopter emergency medical service (HEMS) operation [51]. They performed 41 chest US examinations in 71 patients. Expert sonographers reviewed the images and reached substantial agreement with the providers. Image quality was good or poor in 54 and 46%, respectively. Challenges consisted of the lack of time, limited aircraft space, and, less frequently, the presence of pacer pads. They concluded that in-flight chest US is feasible, has a steep learning curve, and that additional training is needed to improve image quality. Quick et al. found that the diagnostic accuracy of in-flight US for pneumothorax is nearly similar to US in the ED: 91 and 96%, respectively [52].

These reports suggest that PHUS augment the diagnostic capabilities of prehospital aeromedical providers, also when in-flight, and might lead to better outcomes.

#### Hemothorax

No studies with substantial data on the diagnostic performance of PHUS and hemothorax are available. Ketelaars described that PHUS detected one hemothorax in seven cases specifically assessed for hemothorax with 100% accuracy [4]. In a 2007 *best evidence topic report* the authors concluded that ultrasound is a sensitive, specific, and accurate method to detect the presence of hemothorax in trauma patients [53]. A more recent meta-analysis of hospital studies revealed a pooled sensitivity and specificity of 67 and 99% of PoCUS for hemothorax. For radiography, these were 54 and 99% [54]. Therefore, PoCUS for hemothorax may be valuable in both in-hospital and prehospital settings. Future studies might demonstrate the added value of early, prehospital, detection of hemothorax although an early chest tube thoracostomy is rarely required [55]. Still, PHUS yields valuable information to include in the prearrival notification to the receiving trauma center.

#### Diaphragmatic rupture

Diaphragmatic rupture occurs in up to 5% of blunt abdominal trauma patients and may be present despite a negative FAST scan [56]. Ultrasonographic signs may be poor movement (on M-mode) or elevation of the diaphragm, a liver sliding sign (at the right chest wall), subphrenic effusion, or the presence of an intrathoracic spleen or liver [57–59]. In addition, Gangahar introduced *Rip’s absent organ sign* as an indirect marker: nonvisualization of the spleen or heart caused by displacement of abdominal contents anteriorly to these organs [60, 61].

### B—Gastric tube

The only indication for a gastric tube (GT) in the prehospital setting is to relieve gastric distention that is often caused or aggravated by bag-valve-mask ventilation. Traditionally, correct positioning is verified by injecting air in the tube while listening for air bubbles, or by aspiration of gastric contents. These methods are unreliable, especially in the noisy prehospital environment, and the recommended pH measurements and chest X-rays are not feasible [62]. Chenaitia et al. estimated the diagnostic accuracy of PHUS confirming GT placement in 130 prehospital intubated patients, compared to in-hospital chest X-ray. They positioned the probe subxiphoidal in the transverse plane, oriented toward the left hypochondrium to visualize the GT tip in the gastric antrum. Examination time was limited to 1 min. Sensitivity and specificity were 98.3 and 100%. PPV and NPV were 100 and 85.7% [63].

In a follow-up study they added an esophageal view at the anterior neck during and after GT insertion. In case the GT was visualized in the esophagus but not in the stomach, 50 ml of air was inserted. An intragastric position of the tip was visualized or assumed when gastric air entry was observed as *dynamic fogging*: an expanding volume of hyperechoic ‘fog’. Sensitivity and specificity were both 100% compared to in-hospital chest X-ray [64]. When US is only performed after GT insertion, it is as fast as the traditional air insufflation and aspiration method.

### C—Circulation–cardiac arrest

Current European resuscitation guidelines state that there is no doubt that focused cardiac ultrasound (FoCUS)—using specific protocols for US evaluation—has the potential to detect reversible causes of cardiac arrest [12]. FoCUS can help distinguish the PEA type, identify the cause of the arrest, choose a suitable treatment, and make the right decision on cardiopulmonary resuscitation (CPR) termination [65]. In 75% of the patients with pulseless electrical activity (PEA), FoCUS showed coordinated cardiac motion (pseudo-PEA) in a prehospital peri-resuscitation care study [66]. Pseudo-PEA is strongly associated with increased survival compared to a true PEA. Treatable causes were reduced ventricular function (59%), pericardial tamponade (9.8%), a significantly dilated right ventricle (7.8%), and hypovolemia (3.9%) [66]. A return of spontaneous circulation (ROSC) was indeed achieved after pericardiocentesis. Three of five tamponade patients survived to hospital admission.

Similarly, cardiac motion in PEA patients in the ED is positively associated with ROSC. Salen found that in 8 of 11 (73%) patients with sonographic cardiac activity, ROSC was achieved but in none of 23 without cardiac activity [67]. A retrospective analysis of 318 pulseless trauma patients revealed that the survival of pulseless traumatic arrest patients without sonographic cardiac activity is rare [68]. In non-trauma ED patients, cardiac standstill on FoCUS during CPR correlated with death with a PPV of 97.1% and an NPV of 57.1% [69]. However, the timing and the duration of the FoCUS examination could be very important.

Termination of resuscitation (TOR) may be considered in out-of-hospital cardiac arrest patients when these four criteria are met: no ROSC before transport, no shock delivered, no bystander CPR, and an unwitnessed arrest [70]. Goto developed a similar TOR rule: no prehospital ROSC, non-shockable initial rhythm, and unwitnessed by bystanders. Their rule is a > 99% predictor of death within 1 month after out-of-hospital cardiac arrest (OHCA) [71]. Cardiac standstill on initial FoCUS may predict non-ROSC and could be used in the decision for the termination of treatment [67, 72]. However, a 2016 study in non-traumatic OHCA patients undergoing serial FoCUS confirmed ROSC could occur within 10 min after initial cardiac standstill [73]. However, after a cardiac standstill of 10 min or longer, no ROSC occurred. These findings suggest that PHUS might play an important role here: consider TOR after 10 min of sonographic cardiac standstill?

In addition to uncovering treatable causes of cardiac arrest, FoCUS is invaluable in confirming mechanical ventricular capture (as opposed to electrical capture) during transcutaneous cardiac pacing [74].

### C—Shock

Although the cause of shock may not be apparent, FoCUS might guide therapy such as intravenous fluid administration, inotropic therapy, and the choice of destination hospital. FoCUS directly altered treatment in 51% of the cardiac arrest and peri-resuscitation patients in Breitkreutz’s prehospital study [66]. This implies that every hemodynamically unstable patient could potentially benefit from PHUS.

#### Non-traumatic shock

**Fig. 6:**
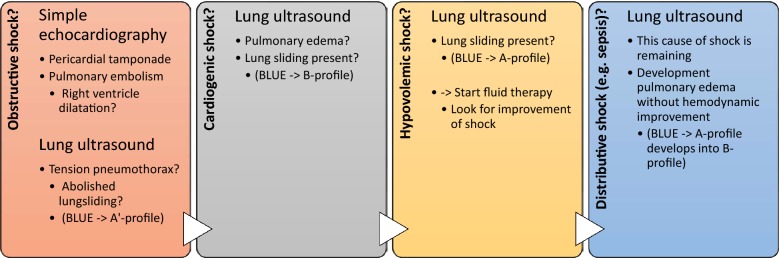
FALLS protocol. This diagram is an adaptation of the work by Dr. Lichtenstein [39]. Firstly, this diagram shows the type of shock the focus is on. Secondly, the type of ultrasound examination is shown. Thirdly, possible diagnoses to consider are shown including their appearance in terms of the BLUE protocol. Every cause of shock is sequentially excluded for expediting the diagnosis of distributive (septic) shock. *FALLS* fluid administration limited by lung sonography, *BLUE* bedside lung ultrasound in emergency, BLUE and the A, B, and A’ profile are explained in Table 1, items 1, 2, and 8, respectively

To evaluate critically ill patients with acute circulatory failure, Lichtenstein devised the fluid administration limited by lung sonography (FALLS)-protocol aimed at reducing the mortality from septic shock [39]. It aims to sequentially rule out (1) obstructive, (2) cardiogenic, and (3) hypovolemic shock for expediting the diagnosis of distributive (usually septic) shock, displayed in Fig. 6. When other causes of shock are eliminated and distributive shock (sepsis) remains, fluid therapy and vasopressors are indicated. Fluid therapy is guided by repeated chest ultrasound based on the appearance of the so-called B-profile as defined in the BLUE protocol (Table 1).

The rapid ultrasound in shock (RUSH) examination involves a three-part assessment simplified as (1) the pump, (2) the tank, and (3) the pipes [75]. *The pump* refers to an evaluation of the pericardial sac, left ventricular contractility, and the relative size of the right ventricle to the left ventricle. *The tank* refers to the determination of effective intravascular volume status by measuring the inferior vena cava (IVC) and assessment of the lung, pleural and abdominal cavity. *The pipes* refer to scanning for an aneurysm or dissection of the thoracic and abdominal aorta, and deep venous thrombosis.

Both the FALLS and RUSH protocol combine familiar US scans proven to be feasible in the prehospital setting. Although we are unaware of any reports, these protocols are potentially valuable in prehospital care.

#### Traumatic shock

**Fig. 7:**
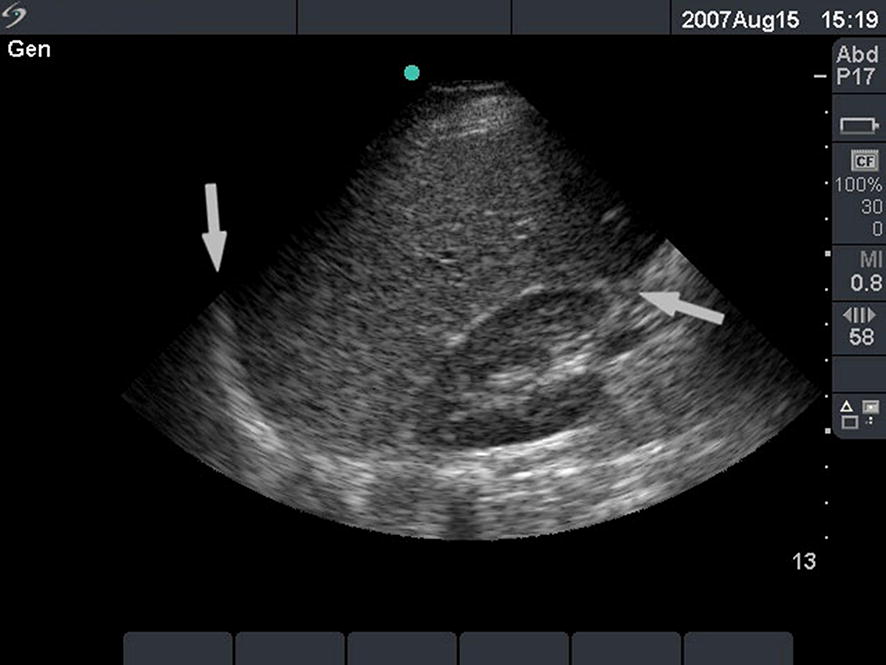
Normal hepatorenal recess. A normal ultrasound image of the hepatorenal recess (Morison’s pouch). A phased-array cardiac transducer was used with the abdominal settings. The left arrow indicates the diaphragm. The right arrow indicates the hepatorenal recess. The liver is shown above this line and the right kidney below

**Fig. 8:**
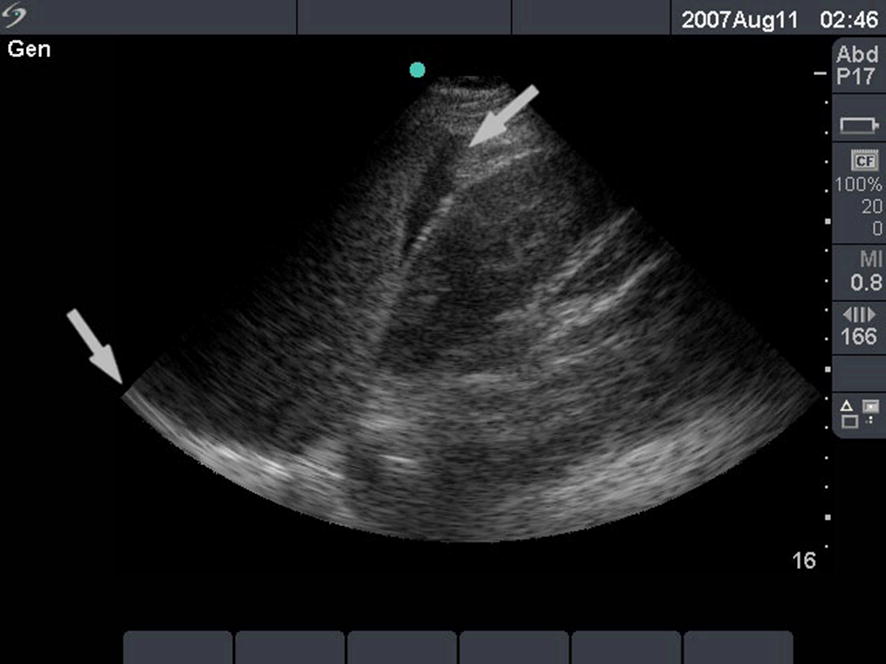
Hemoperitoneum at the splenorenal recess. An ultrasound image of the splenorenal recess (Koller’s pouch). A phased-array cardiac transducer was used with the abdominal settings. The left arrow indicates the diaphragm. The right arrow indicates the splenorenal recess with a hypoechoic collection between the spleen (left) and the left kidney (right). This is the image of free intraperitoneal fluid and is very suggestive for intraperitoneal hemorrhage when encountered in a trauma victim

In traumatic shock, the (extended) FAST protocol may be used to detect a hemoperitoneum. A US image of a normal hepatorenal recess and one with a hemoperitoneum are displayed in Figs. 7, 8. In abdominal trauma, its sensitivity and specificity are comparable between in-hospital and prehospital: 100 and 97.5% in-hospital and 90 and 99% prehospital, respectively [2]. The feasibility and efficiency of the extended FAST were also comparable, with no significant difference in US duration [22].

In shocked blunt abdominal trauma patients, expeditious PoCUS should take a minimum amount of time. Clarke found that mortality increases by 1% for every 3-min delay of a necessary intervention [76, 77]. Unfortunately, false-negative results do occur and they do most frequently in scans performed early in the disease process [22, 78]. Therefore, when FAST is negative it is recommended to repeat the examination every 15 min [2, 77]. Repeated abdominal US scans may lead to a 50% reduction of false negatives [79]. However, a hemorrhage in the retroperitoneum or any solid organ injuries cannot be detected reliably with FAST. So, a negative FAST does not compensate for a high suspicion of abdominal hemorrhage.

A (non) traumatic pneumoperitoneum is almost invariably caused by gastrointestinal perforation. When detected prehospitally, this might steer early treatment and transportation. Sensitivity and specificity of abdominal US for pneumoperitoneum is 85–90% and 100%. In experienced hands it can be as good as CT; an amount as small as 1 ml of free air can be detected [80–82]. Thus, it appears plausible to use PHUS also for this indication.

In their meta-analysis, Stengel et al. concluded that US for blunt abdominal trauma does not decrease the laparotomy rate or mortality. Nevertheless, the number of ordered CT scans decreased by 50%. However, this might reflect a false sense of security due to the low sensitivity of abdominal ultrasound for both free fluid and organ lacerations [7]. Montoya also reported that US led to fewer CT scans. In addition, however, they found a decreased time to appropriate interventions, shortened hospital stay, and decreased use of healthcare resources [78].

### C—Abdominal aortic aneurysm

US is feasible and suitable to assess for an aneurysm of the abdominal aorta in symptomatic patients. An ED study showed a sensitivity and specificity of 100 and 98% [83]. Prehospitally this is also feasible, reported Heegaard et al. Trained ambulance paramedics performed PHUS scans of the abdominal aorta in 20 symptomatic patients. A blinded expert also judged the images and agreed 100% with the paramedics’ judgment [84].

### D—Central nervous system

#### Stroke—transcranial US

Reducing the interval between ischemic stroke and intravenous thrombolysis is associated with reduced mortality, reduced symptomatic intracranial hemorrhage, and higher rates of independent ambulation at discharge and discharge to home [85, 86]. Unfortunately, prehospital delays lead to missed opportunities to initiate treatment within the preferred 90 min after the onset of symptoms [87]. For instance, in an American study, only 38% of patients arrived within 2 h of the onset of their symptoms [88].

Early prehospital detection of ischemic stroke may be beneficial to a favorable outcome. Prehospital caregivers could allocate patients to the most appropriate hospital, provide a prearrival notification, and initiate stroke-specific therapies such as sonothrombolysis and neuroprotective strategies [87, 89, 90]. Intravenous thrombolysis, however, is only administered safely after a CT or magnetic resonance imaging (MRI) scan excludes an intracranial hemorrhage.

Herzberg et al. evaluated the diagnostic accuracy of prehospital neurological examination supported by transcranial color-coded sonography (TCCS) [89]. The TCCS consisted of color-mode visualization and flow measurements in the proximal M-1 segment of both middle cerebral arteries (MCA) to find an occlusion. When desired, they scanned the anterior and posterior cerebral arteries or administered an intravenous ultrasound contrast agent (UCA). Sensitivity and specificity were 95 and 48%; PPV and NPV were 82 and 77% for the prehospital diagnosis of ‘any stroke’ compared to in-hospital CT angiography (CTA) and magnetic resonance angiography (MRA). With appropriate training, telemedicine, and UCAs, these results might still improve [89].

In their prehospital study, Schlachetzki found that 36% of the physicians used microbubbles as a UCA to save time or to increase the diagnostic confidence when temporal window anatomy did not allow an optimal visualization of the MCAs. The sensitivity and specificity of ultrasound for MCA occlusions were 90 and 98%. PPV and NPV were 90 and 98% [90].

Besides arterial occlusions, vasospasm due to aneurysmal subarachnoid hemorrhage may be detected by transcranial US. However, its diagnostic accuracy varies widely depending on the vessel, the diagnostic criteria, and timing [91]. Anecdotal evidence exists on other intracranial pathologies which may be detected by PHUS such as intracranial hematomas and ventricular system enlargement [92, 93].

**Fig. 9:**
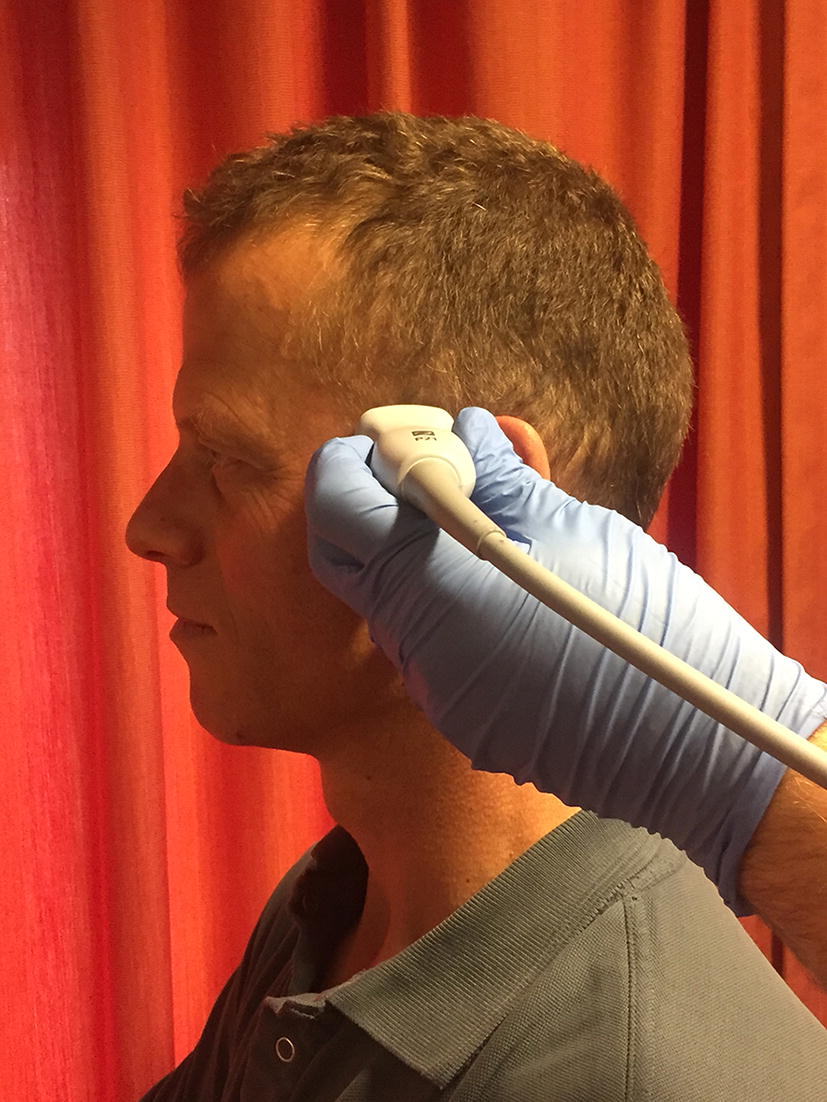
Ultrasound transducer positioned at the temporal window. The ultrasound transducer is positioned at the site where the temporal bone is thinnest and the ultrasound beam is least obstructed penetrating the skull (Reproduced with permission from Rob Stoffels and Yvonne Houben)

The biggest limitation of the application of transcranial US is the ability to obtain US images through the temporal window (Fig. 9). This is the thinnest part of the temporal bone that allows penetration of the US beam at a suitable angle and distance in relation to basal portions of the major cerebral arteries and the circle of Willis. This procedure may be very demanding and requires training and experience. Therefore, transcranial US might not be suitable for every ultrasound-equipped (H)EMS service.

The therapeutic applications of transcranial US in ischemic stroke are discussed in the *interventions* section of this paper.

#### ONSD

Both optic disc edema detected by fundoscopy and an increased optic nerve sheath diameter (ONSD) are indications of increased intracranial pressure (ICP) [94]. The ONSD can easily be measured using US, although the use of a UCA might enhance the recognition of relevant anatomy [95]. ONSD measurements, using a cutoff value of 5.0 mm, have a sensitivity and specificity of 100 and 95% in predicting an elevated ICP compared to CT [96]. Moretti et al. compared US to invasive ICP measurements. Using a cutoff value of 5.2 mm, they found a sensitivity and specificity of 93.1 and 73.9% for an ICP ≥ 20 mmHg [97]. More recently, Maissan et al. measured the ONSD in ICP-monitored traumatic brain injury (TBI) patients before, during, and after routine suctioning of the endotracheal tube leading to a transient ICP rise. With a cutoff value of 5.0 mm they found the sensitivity and specificity to be 94 and 98% for a rise in ICP [98].

Like US in ischemic stroke, the benefit of prehospital ONSD measurements might be to start neuroprotective strategies, to determine the need for neurosurgical care, or to provide a prearrival notification. However, the evidence on prehospital feasibility and benefit is still negligible.

### E—Injuries

#### Fractures

Dulchavsky concluded that non-physicians in the ED (cast technicians) can reliably diagnose orthopedic injury with an accuracy of 94% after a brief PoCUS training. This was more reliable for fractures of the humerus, forearm, femur, and lower leg than for hand and foot fractures, and tendon injuries [99].

Bozorgi et al. evaluated US in 108 ED patients with 158 fractures in the extremities. The overall sensitivity was 68.3%. Sensitivity for femoral fractures and humeral fractures was 100 and 76.2%, respectively. The detection of intra-articular fractures was the most difficult with a sensitivity of only 48% [100].

In the civilian prehospital setting, PHUS for fractures is probably feasible. It is useful for (long) bone fractures in the upper and lower arm, and leg. Advantages in the prehospital setting could be early reduction and splinting, triage, and selecting the best destination provided with a specific prearrival notification.

#### Foreign objects

In the wilderness environment, Paziana described two cases where PHUS was successfully used to aid in the removal of foreign objects from soft tissue [101]. PHUS may determine the exact location and depth of a foreign object despite that some are radiolucent. The location and size of the incision can be determined, and the removal may take place under direct US visualization.

#### Ocular ultrasonography

Besides ONSD measurements, ocular US appears useful and feasible in the prehospital setting. It is useful to diagnose penetrating globe injury, foreign body retention, retinal detachment, vitreous detachment, central retinal artery occlusion, lens dislocation, retrobulbar hematoma, and retinal and vitreous hemorrhage [102, 103]. The eye can be accurately assessed without the need to open the eyelid in case of swelling. An austere environment case report described PHUS helping to diagnose a retinal detachment after a facial gunshot wound. Immediate evacuation was arranged to facilitate appropriate follow-up care [104].

## Interventions

Here, many diagnostic applications of PHUS have been discussed. Besides its diagnostic applications, PHUS has been shown to be potentially valuable in guiding interventions or as a therapeutic intervention in its own right.

### Interventions—airway

Emergency percutaneous cricothyroidotomy may be unsuccessful or produce a tear in the posterior tracheal wall. Siddiqui et al. compared anatomical orientation by either digital palpation or US for performing a percutaneous cricothyroidotomy with the Portex^®^ device. In cadavers in which palpation of the cricothyroid ligament is difficult, US increased the probability of a correct device insertion by 5.6 times and reduced the incidence of laryngeal and tracheal injury from 100 to 33%. A possible disadvantage of US may be the prolonged time to airway insertion [105].

In the emergency prehospital setting, the open cricothyroidotomy is the preferred approach in invasive airway management. Whether a US-guided percutaneous technique should be used in an emergency at all is a matter of debate. Nevertheless, Curtis et al. found a US-guided bougie-assisted open cricothyroidotomy to be a rapid and reliable technique. Cricothyroidotomy was successful in 20 of 21 cadavers, with a median time to completion of 26.2 s [106].

### Interventions—breathing

Medical patients with severe symptomatic pleural effusion might require early pleural aspiration in the prehospital setting. Pleural US is useful in the diagnosis and localization of fluid. US-guided thoracentesis is a safer and more effective method to relieve symptoms than a blind approach [107, 108].

### Interventions—circulation

To guide interventions, PHUS is most frequently used for (central) venous access. It was the second most used PHUS application overall (after assessment of blunt abdominal trauma) in an Australian retrieval team [47]. Intraosseous access is the most appropriate approach in time-critical emergencies. However, for less urgent but difficult to obtain peripheral intravenous access, US guidance is faster and more effective than traditional catheter insertion [109].

Symptomatic pericardial effusion might need prompt treatment in the prehospital setting. US-guided pericardiocentesis under continuous visualization using a multi-angled needle guide was found to be effective, safe, and easy to perform [110].

First described in 1954, resuscitative endovascular balloon occlusion of the aorta (REBOA) is a technique to stabilize patients suffering hemodynamic shock by temporarily interrupting blood flow to non-compressible hemorrhage in the chest, abdomen, or pelvis [111, 112]. In animal studies, REBOA resulted in a 74% mortality risk reduction [113]. After 40 min of occlusion, however, the risks start outweighing the benefits [113, 114]. In 2014, the London HEMS was the first to report a prehospital performed REBOA in a patient with a pelvic fracture resulting in successful hemorrhage control [115].

Chaudery found that the use of US improved the correct placement of REBOA catheters, shortened the time until correct placement, and improved the participants’ confidence in catheter placement of Zone III (infrarenal aorta) REBOA catheters in 20 porcine cadavers [116].

These developments are promising for future prehospital US-guided REBOA hemorrhage control. However, future research is needed on prehospital feasibility, variations in body habitus, and zone I (intrathoracic aorta) placement.

### Interventions—disability

In the aforementioned *disability* section, we highlighted the value of TCCS in diagnosing ischemic stroke. A therapeutic application of ultrasound in ischemic stroke patients is continuous transcranial Doppler (TCD) to enhance the thrombolytic activity of tissue plasminogen activator (t-PA) [117]. In a phase II multicenter randomized trial (CLOTBUST), transducers were applied over the temporal bone in a head frame. The investigators applied TCD (or placebo TCD) on maximum power output continuously for 2 h and simultaneously started intravenous t-PA treatment in all patients. Two hours after starting thrombolysis, recanalization or almost full recovery was observed in 49% in the continuous TCD group versus 30% in the control group. However, clinical recovery after 24 h and outcome after 3 months was similar [117]. In 2014, another analysis of the CLOTBUST trial, including more subjects, revealed 38.6% complete recanalization in the sonothrombolysis group and 17.1% in the intravenous t-PA group [118]. A phase III trial is underway [119]. Tsivgoulis concluded in a meta-analysis that high-frequency ultrasound (both TCD and TCCS) combined with t-PA was associated with a higher likelihood of complete recanalization (pooled OR = 2.99) than t-PA alone [120]. They found no increased risk of symptomatic intracerebral hemorrhage.

Probably, transcranial US combined with microbubbles but without t-PA is effective as well [121, 122]. Microbubbles consist of an injectable aqueous suspension of small (1.5–4.7 µm) bubbles of a high molecular-weight gas that is used as a US contrast agent to improve the visualization of blood vessels [123]. In a meta-analysis, Saqqur indeed concluded that sonothrombolysis with or without microbubbles or t-PA was effective and safe [124]. These findings allow the exploration of early prehospital initiation of sonothrombolysis in suspected ischemic stroke without needing a CT or MRI scan. Hölscher already suggested that PHUS could serve to ‘precondition’ the culprit clot to increase its therapeutic sensitivity to t-PA or neuro intervention while providing neuroprotection for tissue at risk [121].

### Interventions—regional anesthesia

US-guided regional anesthesia is a common technique for providing perioperative pain relief for elective surgical procedures of the extremities. These techniques can also be employed in the prehospital setting to provide effective analgesia for extremity injuries and avoid the side effects associated with the administration of systemic analgesics. For instance, ultrasound-guided femoral nerve blocks effectively provide pain relief in hip fractures [125]. Also, PHUS might facilitate already successful prehospital fascia iliaca compartment blocks [126]. Similarly, Lippert et al. suggested the added value of US-guided nerve blocks to improve pain control in disaster settings [127].

The transversus abdominis plane (TAP) block is an effective technique for pain relief in pelvic fractures and because of its ease and safety it may be applicable in the prehospital setting [128]. Blocking the nerves that supply the anterior abdominal wall relaxes the abdominal wall muscles that will subsequently reduce the traction on the ischium and pubis. The ‘flank bulge sign’ is a direct consequence of this relaxation [129].

### Disaster triage

In a multiple casualty incident (MCI), resources are limited. Triage systems are used to determine treatment priority of the injured patients based on history and physical examination. PoCUS was reported to be valuable in the triage process during several earthquake disasters [130–134]. Stawicki proposed a protocol that integrates some common PoCUS applications to evaluate the chest, abdomen, vena cava, and extremities as an adjunct to acute triage (CAVEAT) and to be executed during the secondary survey [135]. The protocol will take approximately 5 min longer than a traditional FAST scan. It is explained in more detail in Table 2. Although the merits of its component parts have been described extensively, the benefit of the protocol is yet to be established.

**Table 2:** The CAVEAT protocol. Stawicki et al. [135]

Urgency	Step	Examination	Focus on	Looking for
CAVEAT protocol				
Primary assessment (mandatory)	1	Evaluation of the pleura	Chest	Pneumothorax
	2	Complete FAST examination	Abdomen Costophrenic recesses	Hemoperitoneum Hemothorax
	3	Inferior Vena Cava assessment	Collapsibility index	Volume depletion
Secondary assessment (optional)	4	Upper- and lower extremities	Long bones; regions of pain, tenderness, or deformity	Major fractures eligible for more accurate reduction and stabilization Fractures to prioritize utilization of radiographic resources, or achieve even more accurate triage

*CAVEAT* sonographic evaluation of the chest, abdomen, vena cava, extremities for acute triage, *FAST* focused assessment with sonography for trauma

This table shows the suggested order of examinations in the CAVEAT protocol. Specific components may depend on the operators’ skill level and on the individual patient’s injuries

## Future applications

### Sonothrombolysis

As we have discussed in the *interventions* section, early prehospital sonothrombolysis in ischemic stroke patients might be safe and effective. The CLOTBUST investigators have developed a hands-free headframe containing 18 ultrasound transducers positioned at the temporal occipital bone windows to deliver operator-independent ultrasound energy directly to the culprit clot. It was successfully applied to and well tolerated by 15 volunteers and is currently evaluated in stroke patients [136]. It may facilitate and enhance early thrombolysis because of its portability and that no formal ultrasound training is needed.

### Telemedicine

With improving data communication technologies, telemedicine is a promising technique for remotely evaluating ultrasound clips acquired by less experienced operators. They might even be coached in real time supported by remotely operating the ultrasound device settings in complex scenarios [137]. Kolbe introduced a PoCUS curriculum in a one-room medical clinic in rural Nicaragua. Despite limited resources, after the first introduction the ultrasound instructors used telemedicine to remotely view the ultrasound images in real time [138]. In 2016, Kirkpatrick demonstrated the feasibility of remotely telementoring ultrasound-naïve firefighters using trauma ultrasound for free fluid detection on a phantom [137]. Remote telementored ultrasound was feasible to coach untrained and inexperienced nurse practitioners to assess patients for pneumothorax immediately after removal of their tube thoracostomy [139]. Rubin demonstrated the feasibility of remote review and interpretation of TCD and carotid ultrasound data in healthy volunteers dubbed “teleneurosonology” [140].

Integrating telemedicine concepts in PoCUS-enhanced disaster triage might be promising and feasible in the light of progressing technological advancements.

### Wearable US

Mierzwa developed a flat and flexible 5 MHz US probe designed to wear on a fingertip to aid in US-guided vascular access, for instance. The device can be configured as a linear or curvilinear transducer array and it can be mounted directly onto the body as an adhesive patch or wearable device. They speculate on many applications such as point-of-care imaging, combat casualty care, ultrasound therapy, and patient monitoring [141]. A specific prehospital application might also be a US patch for continuous cardiac visualization during cardiopulmonary resuscitation.

### Assessment of intraosseous needle position

Tsung demonstrated the feasibility of US to determine the location of an intraosseous needle in six resuscitation cases. He argues that every intraosseous access should be verified with color Doppler because a correct position cannot be accurately confirmed by the aspiration of blood, blood on the stylet tip, the needle being firmly in place, or the absence of soft tissue extravasation [142].

### Predicting outcomes in resuscitative thoracotomy (RT)

In some countries, a prehospital or ED resuscitative thoracotomy (RT) is performed on patients with a penetrating (sometimes also blunt) thoracic injury decompensating into cardiac arrest. The goal is to treat a cardiac tamponade or major injuries of the heart, control intrathoracic bleeding, clamp the thoracic aorta, or perform direct cardiac massage or defibrillation. The RT is an invasive and last-resort treatment. Inaba found that FoCUS was a predictor of futile care in these patients [143]. In 187 RT patients, only 6 survived and 3 were eligible organ donors. All survivors and organ donors had visible cardiac motion before RT was performed. If no cardiac motion or pericardial effusion on US was observed, the survival was zero. Thus, utilizing US would have avoided a considerable number of RTs that were ultimately futile [143]. Because of these findings, PHUS would be a valuable addition to prehospital RT protocols.

### US-guided cannulation for extracorporeal life support (ECLS)

Lamhaut concluded in 2013 that prehospital implementation of ECLS by non-surgeons was safe and feasible [144]. Four years later their group described a case of ECLS cannulation in the Louvre museum in Paris in which they used a hybrid surgical/Seldinger technique [145, 146]. Another future PHUS application might be US-guided percutaneous ECLS cannulation that may be easier, faster, and less invasive. It could be complemented by (contrast enhanced) echocardiography to verify correct placement of the venous catheter tip [147, 148].

## Challenges of prehospital ultrasonography

PHUS is subject to specific challenges in the prehospital environment: ambient lighting, confined space, extremes of temperature, precipitation, dressings, splints, and rapid transport times [3, 6].

Diagnostic ultrasound is generally considered harmless. However, it may heat up tissue depending on these factors: exposure duration, the acoustic output, and tissue characteristics. For instance, some unique properties of the eye such as high absorption of ultrasound and the absence of cooling blood supply may cause the lens to heat up faster than other tissues [149, 150]. Therefore, this has to be considered in ocular ultrasound or ONSD measurements.

PHUS is used by nonradiologists mainly to answer simple yes/no questions and to guide treatment decisions. Sensitivity for solid organ injuries is low and small quantities of blood early in the post-injury phase may be missed. Traumatic aortic pathology cannot be detected by chest or abdominal US; therefore, PHUS is not a valid replacement for CT angiography in patients subjected to high-energy thoracic trauma [78]. False negatives will occur; therefore, negative findings should not indicate a final exclusion of diagnoses [3]. Thus, for some indications, the sensible choice might be to use PHUS only as a ‘rule-in’ tool not to be falsely reassured by (false) negative test results.

Another concern of PHUS is the potential delay in treatment. In general, a slight delay might occur when PHUS is performed on-scene. However, delays are non-existent when performed in parallel with other procedures, while in-flight, or during ground ambulance transport. Busch found the median PHUS duration to be 2.5 min (range 1–3) [13]. For a range of PHUS examinations, Hoyer measured a mean of 1 min 54 s, decreasing to 56 s during the 3-year study period [3]. In their review, Jørgensen et al. reported a delay of 0–6 min. Examination time depends on the protocol and the results: positive findings will reduce the examination time [5].

Although a slight delay may occur, this might easily be outweighed by the advantage of improved diagnostic and therapeutic accuracy, and the potential time gains by transporting the patient directly to the most appropriate hospital.

## Conclusions

We have provided a comprehensive summary of the literature on prehospital applications of diagnostic and therapeutic ultrasound structured according to the ABCDE approach. Also, we have highlighted in-hospital PoCUS procedures that appear useful and plausible for prehospital use, current challenges in PHUS, and potential future applications. It may be commendable to revise this review in the near future when, undoubtedly, additional useful PHUS applications will have emerged.

Improvements in portability, quality, and price of handheld ultrasound systems add to the accessibility and its feasibility for prehospital use. PHUS improves the diagnostic capabilities of prehospital health-care providers and might improve treatment decisions, prearrival notifications, and transport mode and destination. As new PoCUS techniques and applications are being researched, new protocols are being tested for diagnostics, procedural guidance, and therapeutic use.

However, prehospital caregivers should unabatedly be aware of the limitations of PHUS. The time investment will not always pay off and diagnostic accuracy is not perfect. Diagnostic accuracy is quite dependent on training and experience of the providers.

The diagnostic and therapeutic possibilities of PoCUS are increasing. With promising techniques, such as sonothrombolysis in ischemic stroke, we are bringing the hospital-level medical care to prehospital patients to an ever-increasing extent [119].

## Additional file

**Additional file 1: Video 1.** Lung point.

## Abbreviations

ABCDE: airway, breathing, circulation, disability, exposure/environment
ADHF: acute decompensated heart disease
BLUE: bedside lung ultrasound in emergency
BNP: B-type natriuretic peptide
CAVEAT: chest, abdomen, vena cava, and extremities as an adjunct to acute triage
COPD: chronic obstructive pulmonary disease
CPAP: continuous positive airway pressure
CPR: cardiopulmonary resuscitation
CT: computed tomography
CTA: CT angiography
ECLS: extracorporeal life support
ED: emergency department
eFAST: extended FAST
EMS: emergency medical service
ETT: endotracheal tube
EVLW: extravascular lung water
FALLS: fluid administration limited by lung sonography
FAST: focused assessment with sonography for trauma
FoCUS: focused cardiac ultrasound
GT: gastric tube
HAPE: high-altitude pulmonary edema
HEMS: helicopter emergency medical service
ICP: intracranial pressure
IVC: inferior vena cava
LCI: lung ultrasound; cardiac ultrasound; inferior vena cava measurements
MCA: middle cerebral artery
MCI: multiple casualty incident/mass casualty incident
M-mode: motion mode
MRA: magnetic resonance angiography
MRI: magnetic resonance imaging
NPV: negative predictive value
OHCA: out-of-hospital cardiac arrest
ONSD: optic nerve sheath diameter
PAOP: pulmonary artery occlusion pressure
PEA: pulseless electrical activity
PHUS: prehospital ultrasound
PoCUS: point-of-care ultrasound
PPV: positive predictive value
REBOA: resuscitative endovascular balloon occlusion of the aorta
ROSC: return of spontaneous circulation
RT: resuscitative thoracotomy
RUSH: rapid ultrasound in shock
TAP: transversus abdominis plane (block)
TBI: traumatic brain injury
TCCS: transcranial color-coded sonography
TCD: transcranial Doppler
TOR: termination of resuscitation
UCA: ultrasound contrast agent
US: ultrasound
